# An exploratory qualitative pilot study assessing treatment-seeking behavior for generalized anxiety symptoms among people living with HIV/AIDS in Tanzania

**DOI:** 10.1371/journal.pmen.0000348

**Published:** 2025-10-06

**Authors:** Frank Kiwango, Carl Mhina, Lyidia Vedasto Masika, Editruda Gamassa, Florian Emanuel Ghaimo, Neema Allen Ng’unda, Nyasatu G. Chamba, Charles Muiruri, John Bartlett, James S. Ngocho, Blandina T. Mmbaga

**Affiliations:** 1 Department of Mental Health and Psychiatry, Kilimanjaro Christian Medical Centre, Moshi, Kilimanjaro, Tanzania; 2 School of Medicine, KCMC University, Moshi, Kilimanjaro, Tanzania; 3 Kilimanjaro College of Health and Allied Sciences, Moshi, Kilimanjaro, Tanzania; 4 Department of Population Health Sciences, School of Medicine, Duke University, Durham, North Carolina, United States of America; 5 Kilimanjaro Clinical Research Institute, Moshi, Kilimanjaro, Tanzania; 6 Department of Internal Medicine, Kilimanjaro Christian Medical Centre, Moshi, Kilimanjaro, Tanzania; 7 Department of Internal Medicine, KCMC University, Moshi, Kilimanjaro, Tanzania; 8 Duke Global Health Institute, Duke University, Durham, North Carolina, United States of America; 9 School of Public Health, KCMC University, Moshi, Kilimanjaro, Tanzania; University of Luxembourg: Universite du Luxembourg, LUXEMBOURG

## Abstract

People living with HIV/AIDS (PLWHA) are more likely to experience significant generalized anxiety symptoms compared to the general population, which require both pharmacological and psychological treatment. There is limited evidence on the specific facilitators and barriers to treatment-seeking behaviours for significant generalized anxiety symptoms among PLWHA in Kilimanjaro region. This study aims to determine facilitators and barriers to treatment-seeking behaviours for significant generalized anxiety symptoms among PLWHA in Kilimanjaro region. This exploratory pilot study employed a qualitative design to assess facilitators and barriers to treatment-seeking for generalized anxiety symptoms among PLWHA in Kilimanjaro region and used the Generalized Anxiety Disorder-7 (GAD-7) screener to identify significant generalized anxiety symptoms in adult PLWHA who were followed up for three months. Thematic framework analysis was employed to identify and interpret patterns in treatment-seeking behaviors. Among 348 PLWHA, only 8.6% (30 PLWHA) had screened positive for significant generalized anxiety symptoms. Among 30 with significant anxiety symptoms, 6 (20%) PLWHA attended mental health services, 15 (50%) PLWHA did not attend, and 9 (30%) PLWHA were lost to follow-up. Facilitators identified were having mental health awareness, support from family and friends, and having good customer service in mental health clinics. Economic burdens, few mental health services, and stigma were potential barriers to treatment seeking for significant generalized anxiety symptoms. Addressing both facilitators and barriers to treatment-seeking for significant generalized anxiety symptoms among PLWHA, including increasing awareness, reducing stigma, improving access to services, and providing strong social support, is essential for enhancing mental health care.

## Introduction

Anxiety disorders are prevalent worldwide and significantly impact individuals’ quality of life and well-being [1]. For specific populations, like people living with HIV/AIDS (PLWHA), anxiety can complicate both physical and mental health, creating unique challenges [2,3]. Various pharmacological and psychological interventions have been developed to manage these symptoms [4,5]. Pharmacological interventions include the use of medications such as selective serotonin reuptake inhibitors (SSRIs), selective norepinephrine reuptake inhibitors (SNRIs), tricyclic antidepressants (TCAs), and benzodiazepines [6,7], while psychological interventions include Cognitive Behavioral Therapy (CBT), interpersonal therapy, mindfulness-based therapy, and psycho-education [5,8,9]. While both pharmacological and psychological interventions are evidence-based and practiced across high- and low-income countries, including Tanzania [8,10], their contextual applicability remains underexplored. For instance, the availability and consistent supply of SSRIs in Tanzania is uncertain, and there is limited evidence on the accessibility, adaptation, and effectiveness of structured psychotherapies such as CBT within routine clinical settings for PLWHA.

To be able to access these interventions, there are limited reported facilitators in treatment-seeking for significant anxiety symptoms among PLWHA, such as the presence of social support from family members and friends or the use of peer support groups, which often provide emotional and economic support [11]. Also having a good rapport between the health care providers and clients significantly impact their treatment-seeking behavior hence the client will feel they can trust their healthcare providers, they are more likely to discuss their mental health concerns including significant generalized anxiety symptoms and this can be done by integrating the mental health services in care and treatment clinics (CTC) as observed in Cameron [12].

However, in the general population, several facilitators are identified as having mental health awareness, availability of mental health services with professional mental health experts, presence of health insurance coverage, or low cost of mental health services play an important role in encouraging individuals to seek treatment for significant generalized anxiety symptoms as reported [13–15]. In Tanzania, no retrievable studies have explored the facilitators in seeking treatment for significant generalized anxiety symptoms in PLWHA.

Despite the usefulness of interventions, PLWHA face numerous barriers in accessing treatment for significant generalized anxiety symptoms. Individual barriers include economic challenges such as the cost of medications, consultations, and transportation, as highlighted in a literature review [16]. These issues suggest the need for integrating mental health services into HIV care to improve outcomes, a point also supported by studies in Sub-Saharan Africa (SSA) [17,18].

Stigma is another major barrier, as fear of discrimination or judgment by healthcare providers and society can deter individuals from seeking mental health care, as seen in China and Indonesia [4].

Barriers remain, especially for those with generalized anxiety disorder. A systematic review in SSA found that limited funding and resources constrain access to medications, as national health budgets often prioritize infectious and physical health needs over mental health. In addition, the shortage of medical and mental health professionals in LMICs further limits engagement in care, with untreated anxiety contributing to poor medication adherence [17,18]. Finally, long distances to healthcare facilities, especially in SSA, where there are few psychotherapy centers, also pose a significant barrier [4,5,19].

Despite the availability of effective interventions for significant generalized anxiety symptoms, limited studies have explored the treatment-seeking behaviors among PLWHA in Kilimanjaro region. This study aims to explore the treatment-seeking behaviors among PLWHA in Kilimanjaro region experiencing significant generalized anxiety symptoms by identifying barriers and facilitators. We hypothesize that facilitators such as social support and barriers such as resource limitations, workforce shortages, and stigma significantly influence treatment-seeking behaviors among PLWHA. By uncovering these dynamics, the study will provide valuable insights to inform strategies that enhance mental health care for PLWHA, in line with national mental health and HIV care policies, and contribute to improving mental health interventions in Tanzania.

## Methods

### Study design and setting

This was an exploratory pilot study that employed a qualitative technique to assess treatment-seeking behaviors among PLWHA who screened positive for significant generalized anxiety symptoms. This study was conducted in Moshi municipality from October 2024 to December 2024.

The study involved CTCs of a zonal hospital, namely Kilimanjaro Christian Medical Center (KCMC) and Mawenzi Regional Referral Hospital (MRRH). These are the only health facilities that offer mental health services such as psychotherapies, nursing services, and mental health consultations by trained medical staff. The facilities are located at Moshi municipality in Kilimanjaro region, with a total of 19 CTCs allocated from primary to tertiary-level health facilities [20]. Kilimanjaro has a population of 230,784 people characterized by a mixture of rural and urban lifestyles [21].

### Study population

The study included all adults (≥18 years) who were screened for significant generalized anxiety symptoms, and then those who screened positive were followed up. Outpatient clients registered to receive CTC services and have received antiretroviral therapy (ART) for more than 6 months.

### Sample size calculation

The sample size was calculated using Cochran’s formula. We used the prevalence rate of 37.8% from a Tanzanian study by [22], which explored anxiety among adults living with HIV/AIDS in Tanzania.

### Sampling procedure

From the municipal CTC2 database, 2,645 and 3,883 clients were registered at KCMC and MRRH, respectively. The number of clients enrolled in each facility was determined by the probability proportional to the size of each facility using the ratios of 0.4:0.6, respectively. Thus, 139 and 209 clients were enrolled from KCMC and MRRH, respectively, meeting the minimum sample size of 348.

The study team used a systematic random sampling approach to select and enroll participants at study facilities by generating a random number every morning (< 19) for both facilities to identify the first client to be enrolled. The next participant enrolled was after every 4th client for both KCMC and MRRH facilities.

**Fig 1:**
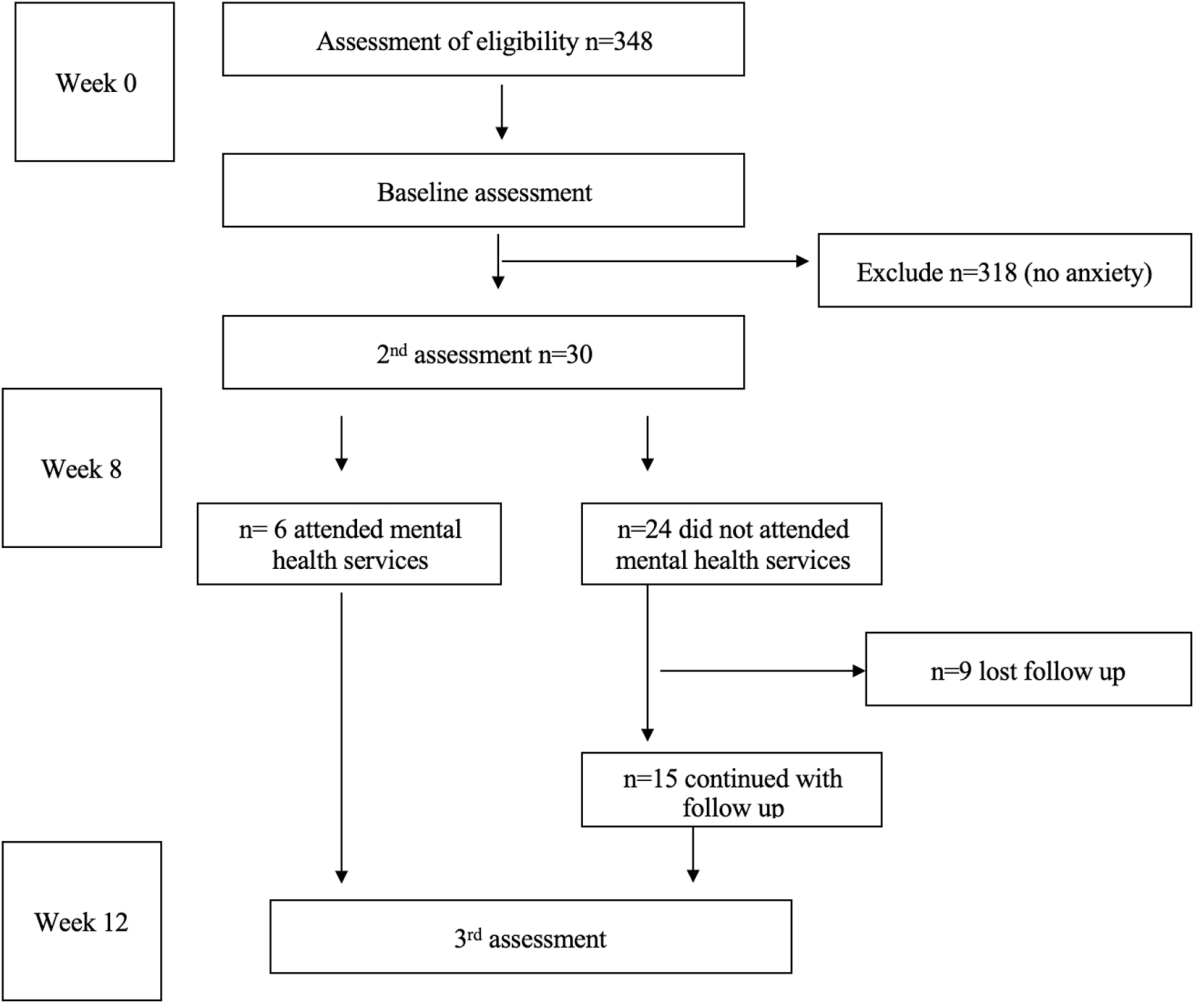
Participants flow chart.

We screened a total of 348 clients across the CTCs using the GAD-7 screener. Of these, 30 clients scored ≥10, indicating significant generalized anxiety symptoms, and were enrolled in the in-depth interviews phase. As such, the sample size of 30 was convenient based on the number of people who had significant anxiety symptoms. This sample size aligns with the acceptable norms for a pilot study aiming to assess usability and process outcomes [23,24]. They were followed for 12 weeks to explore the barriers and facilitators of treatment-seeking behavior. Participants had 3 assessments, namely the baseline assessment, the second assessment, and the third assessment during the follow-up period. A baseline assessment was done at 0 weeks aimed at determining those who will be enrolled for follow-up. The second assessment was done at 8 weeks, and the third assessment was done at 12 weeks. The last two assessments were aimed at assessing the barriers and facilitators of treatment-seeking for mental health services among PLWHA in Moshi municipality-Kilimanjaro region (Fig 1).

### Ethics approval and consent to participate

The KCMC Research Ethics Committee provided the ethical clearance with Certificate number: 2712, while permission to conduct the study was sought from the Municipal Health Office and Medical Officers in Charge of the respective CTC clinics. All participants were verbally informed about the research and provided with information on what participation would entail and the potential risks and benefits. Participants were then asked to read the consent form and sign it to ensure written informed consent was attained from those agreeing to participate**.** Those who were diagnosed with clinically significant anxiety symptoms (GAD-7 ≥ 10) were enrolled in the study and referred to the mental health and psychiatric clinic of their respective or nearby facilities.

### Measures

**Generalized Anxiety Disorder-7 (GAD-7):** The GAD-7 screener assessed the symptoms and severity of generalized anxiety disorder. The GAD-7 is a 7-item questionnaire that asks for experiences with anxiety symptoms in the past two weeks. The total score (range 0–21) is calculated by assigning scores of 0–3 to the response options of “not at all,” “several days in the past two weeks,” “more than half the days in the past two weeks,” and “nearly every day in the past two weeks,” respectively. The cut-off scores for probable anxiety was ≥ 10, with good internal consistency and Cronbach’s alpha of 0.82. The Swahili version has been validated in Kenya among adults with HIV/AIDS [24].

### Data collection procedures

The data collection tools were administered in Kiswahili language by the Principal Investigator (PI), Co-Principal Investigators, and research assistants by using face-to-face interviews. The study team used a random sampling approach to select and enroll participants at each study facility and generated a random number every morning for each facility to identify clients to be enrolled at each facility. We briefly introduced the study before administering the interviews in the Kiswahili language. Participants who consented were enrolled, and the interviews were conducted in private consultation rooms. Participants were assured of the confidentiality of the information they provide as the questionnaire contained a unique number and not the client’s name, assuring that the information they provide cannot be linked to them personally in reports, and the information collected on forms will be stored in a locked cabinet before being entered on a password protected computer that is accessible only to the research team.

In-depth interviews were conducted with the study participants about their experiences of treatment-seeking behaviours. Topic guides were developed by the study team, which included open-ended semi-structured questions and included topics such as barriers and facilitators of treatment-seeking behaviours among participants. The following topics were explored: Personal factors such as mental health history, motivation, and awareness, social support, cost, clinic environment, and stigma.

All these procedures did not interfere with their clinic activities, and after completion of the interview, participants were helped to navigate the clinic activities. No compensation was provided in this study.

### Pre-testing of tools

Before the main data collection, the interview guide was pretested with 5 individuals who closely mirrored the characteristics of the study population. The pretest interviews aimed to assess the clarity, relevance, and cultural appropriateness of the questions. Based on feedback, minor adjustments were made to the wording and the addition of context-appropriate probes. Pretest responses were not included in the final analysis.

### Data analysis plan

Data was collected by using RedCap software version 5.27.2. Data was cleaned to check for missing variables and duplicates.

This study adopts an interpretivist epistemological stance, recognizing that participants’ experiences and perceptions of treatment-seeking behaviours for significant anxiety symptoms are shaped by social, cultural, and contextual factors.

We employed the framework method described by Ritchie and Spencer [25] for thematic analysis, given its suitability for applied health research. After transcription and translation, transcripts were reviewed for familiarization, and initial codes were developed inductively from a subset of interviews. A preliminary codebook was created based on these codes and memo summaries. The entire dataset was then systematically indexed and organized into a matrix to facilitate comparison across cases and themes. Coding was performed using QDA Miner Lite by two independent researchers, with discrepancies resolved through discussion to enhance reliability. Inter-coder reliability was established by double-coding a subset of transcripts and calculating agreement, which demonstrated consistency in coding. We ensured analytical rigor through regular team debriefings, memo-writing for reflexivity, and peer validation of emerging themes. Skipped or unanswered questions were noted as “no response” in transcripts and retained in the analysis without imputation, as non-responses were considered meaningful in reflecting sensitivity or discomfort related to anxiety or HIV. Data Quality Assurance (DQA) procedures were applied throughout the process to maintain accuracy and consistency. Data saturation was considered achieved by the 18th interview, when no new codes emerged. The thematic content framework approach allowed us to systematically interpret patterns related to treatment-seeking behaviors using an inductive content analysis strategy.

## Results

A total of 348 participants were screened in the study. Of the total, only 8.6% (30/348) reported high significant anxiety symptoms. Among 30 with significant symptoms, 20.0% (6/30) attended mental health services up to the third assessment. 80% (24/30) did not attend mental health services, and out of 24 who did not attend the mental health services, 62.5% (15/24) continued up to the third assessment, and 37.5% (9/24) were lost to follow-up. Reasons for loss of follow-up included incorrect contact numbers, poor mobile connectivity, especially those residing in peri-urban areas where network coverage is unreliable, and in some cases, calls could go through but with no response despite being contacted several times.

### Facilitators on seeking mental health services for significant generalized anxiety symptoms among PLWHA

The study’s findings revealed several recurring themes from the IDI. These themes included awareness of mental health issues, social support, presence of role models, and service delivery at the clinics. To enhance the clarity of these findings, we present relevant participant quotes that illustrate each theme.

### Personal factors (Mental health history, motivation, and awareness)

Many participants mentioned that personal experiences with anxiety, as well as awareness of mental health issues, influenced their decision to seek help. For some, the decision was prompted by experiencing anxiety symptoms and recognizing the need for support. Participants with a previous history of mental health challenges were more inclined to seek help sooner. A man said:

*“Well, initially, when I first got this problem, I went to the hospital... it was easy for me to go to the hospital because of this issue.”* **IDI10**

Another one added:

*“As for my motivation, it is simply to improve my health and overall life, because anxiety affects my work and my relationships with others. That is my motivation” IDI13*

### Support from family, friends, and support groups

Support from family, friends, and support groups played a significant role in participants’ decision to seek treatment. Encouragement from family, particularly close friends, and participation in support groups were frequently mentioned as motivating factors. Support groups provided a space for individuals to share experiences and receive guidance on managing both mental health and HIV. One participant reported his close friend encouraged him to attend the clinic and seek help:

*“One of my close friends encourages me a little... He’s the one who motivated me to even start taking steps to seek help.”* **IDI06**

### Role models and motivation

Role models, either from support groups or personal figures such as family members, served as motivation for attending mental health services regularly. Participants mentioned how the example set by others encouraged them to continue treatment and stay committed to mental health care. Our participant reported:

*“Many people encourage me. So, because of that, they attended and received help. Now, that motivates me to go.”* **IDI14**

And another one added:

*“My biggest role model is my father. His words of endurance have greatly encouraged me to follow through with treatment.”* **IDII0**

### Community and health service environment

Access to mental health services was often influenced by the local health service environment and community attitudes. Participants mentioned that health workers who were kind and supportive made it easier to seek treatment, while those who were rude or dismissive discouraged further visits. The community’s understanding of mental health also impacted individuals’ willingness to seek help.

*“In health services... it depends on the day you go and how it turns out. Some health workers... they receive you well... if you get a good day, it’s great.”* **IDI14**

*“If you show emotional vulnerability, it is often taken badly... This creates a fear of openly participating in my mental health challenges.”* **IDI08**

### Barriers on seeking mental health services for significant anxiety symptoms among PLWHA

#### Stigma and societal perceptions.

Stigma surrounding mental health, particularly regarding gender roles and societal expectations, was a common challenge reported by many participants. A man reported:

*“But it’s all about stigmatizing. I get worried when I get out of our clinic. Then I go to another clinic, I may meet maybe someone I know so it’s getting harder for me to do those mental health services, even though I went but it’s getting very hard to follow because with my clinic, when I get in then out …. I am done, but if I start going somewhere else, I’m afraid that someone is going to be following me* ***”* IDI I2**

And another man added:

*“Just the way society sees a man needing mental health help... It creates an image that, to some extent, becomes a challenge.”* **IDI 1**

Another interviewee reported feeling ashamed of having a mental health condition and having no one who understands what he is going through:

*“Aaah! Aaah! I faced a challenge; this anxiety was really troubling me. But after you explained to me that I should see a mental health specialist, that’s when I realized that it was a condition like any other and that I could find a solution. Before that, I used to feel ashamed to talk about my mental health struggles, especially because people didn’t take it seriously. They would just say, “These things are normal, they will pass.” However, after seeing how my condition was affecting my life and my ability to work because I was constantly anxious I recognized the importance of seeking mental health services.”* ***IDI3***

#### Cost and accessibility.

The financial burden of accessing healthcare was a significant barrier. For many, the cost of transportation to the hospital, combined with the lack of insurance, made it difficult to afford regular visits.

*“Mmh, there are costs involved, you know. This challenge is not like other illnesses such as malaria or physical pain. So, when you add the financial burden, a person may see it as a barrier and fail to recognize the importance of seeking care. Also, mental health specialists are very few, even in major hospitals. On top of that, there is stigma in healthcare facilities, which can lead to fear, feeling judged, and not being taken as seriously as other patients.”* **IDI 1**

*“Since I don’t have insurance... I can’t speak well for those who have insurance. But for us who don’t have insurance, it becomes a challenge. So, you suffer until you have money, then you can see the doctor.”* **IDI 04**

#### Shortage of providers and overcrowding.

Another barrier mentioned was the shortage of mental health service providers and overcrowding in clinics. Many participants reported that the high demand for mental health services meant that they had difficulty accessing timely care. It was reported:

*“There are not enough service providers... and the high number of people needing those services... it creates challenges in accessing them.”* **IDI 7**

#### Bureaucratic and structural challenges.

For some participants, navigating bureaucratic processes, including opening medical files or dealing with insurance issues, was a barrier. Time-consuming administrative tasks delayed or discouraged them from seeking help.

*“Time and bureaucracy... these hinder my access to those services such as the hassle of opening a file... it becomes a challenge, it takes too much time to open a file especially when you want to start service in another department “* ***IDI 03***

## Discussion

In this study, various facilitators and barriers were identified in seeking treatment for significant generalized anxiety symptoms among PLWHA. Facilitators included personal experience and awareness of anxiety symptoms, support from family and friends, and the attitude of mental health providers toward PLWHA.

Some PLWHA described how awareness of mental health influenced their decision to seek help. This suggests that awareness may play a role in being able to recognize the symptoms of significant anxiety earlier, hence encouraging individuals to seek professional support, such as from psychiatrists or psychologists, where a previous study in Ireland highlighted that lack of knowledge on mental health disorders, such as anxiety, can lead to delayed treatment seeking among PLWHA [11].

Social support from family and friends was positively associated with treatment-seeking behavior for significant anxiety symptoms among PLWHA. Studies from Europe and China showed that emotional and economic support from close contacts, such as family members and friends, plays a significant role in seeking help for anxiety symptoms [11,26]. This supportive environment fosters long-term accountability and encourages treatment adherence, making individuals feel more comfortable in seeking care and coping with HIV. In the Tanzanian context, family members often serve as the individual’s immediate support base in seeking mental health care. This support is not only emotional, but also practical and financial [3]. Family and close friends commonly accompany individuals to health facilities, assist with transport costs, and offer encouragement to seek professional help. This strong sense of communal responsibility reflects the collectivist nature of Tanzanian society, where health is often seen as a shared concern rather than an individual matter.

The attitude of healthcare providers toward PLWHA with significant anxiety symptoms was another facilitator. This can be explained by the fact that a welcoming and non-judgmental attitude of healthcare providers (HCPs) creates a comfortable environment, allowing individuals to openly share their concerns [27]. This builds trust between PLWHA and HCPs, promoting continued engagement in treatment. This finding is supported by the study from Cameroon, where HCPs with better coordination and communication in managing referrals from HIV clinics to mental health clinics were needed [12].

Apart from the discussed facilitators, there are barriers to treatment-seeking of significant generalized anxiety symptoms that were identified including economic challenges, shortage of mental health services, unintegrated services, and stigma and societal perceptions.

Economic constraints were a significant barrier to seeking mental health care among PLWHA with significant anxiety symptoms. This finding is consistent with other studies [16,17] where financial struggles were identified as a key factor limiting access to care. In our context, clients often have to travel long distances to reach the health facilities that offer mental health services, especially for clients who reside in peri-urban areas, since many of the facilities are located in urban areas. Also, this financial burden was compounded by the lack of comprehensive health insurance coverage in Tanzania. While some participants were enrolled in the National Health Insurance Fund (NHIF), these schemes often do not cover the cost of some of the psychotropic medications or psychotherapies. As a result, out-of-pocket expenses remained high. These economic challenges led some individuals to delay or avoid seeking care altogether, which may worsen both mental and physical health outcomes over time.

Also, the shortage of mental health professionals and services, particularly in rural or underserved areas, was another barrier. This led to overcrowded services, combined with a limited number of qualified practitioners, leading to long wait times and difficulties in accessing care [17,18]. The concentration of mental health services in urban areas further exacerbates the challenge, making it difficult for PLWHA in remote locations to receive timely treatment.

Unintegrated health services posed as an additional barrier. PLWHA often had to go through repetitive processes, such as opening new files and undergoing repeated assessments when seeking mental health care, which increased costs and time spent on consultations. This fragmentation of care creates delays in accessing treatment, discouraging individuals from seeking help. This is evident from reviews done by [18] and [28].

Stigma related to both mental health and HIV/AIDS was identified as a significant barrier to treatment seeking among PLWHA. Participants feared being labeled as “insane” or “weak,” which discouraged them from disclosing their emotional struggles or seeking mental health services. This fear is particularly strong in Tanzanian communities, where mental illness is often misunderstood or linked to spiritual causes, and personal issues are easily shared within communities [29]. The double stigma of living with HIV and experiencing mental health symptoms created a deep sense of shame and fear of social exclusion. This highlights how societal perceptions and cultural beliefs around mental health significantly influence care-seeking behavior in Tanzania, which can increase anxiety, making individuals reluctant to seek care. The findings are similar as those reported by [4,11].

An unexpected finding was the relatively low clinic attendance among some participants. Barriers such as transportation costs, having multiple clinics, and stigma likely contributed to this pattern. Recognizing these challenges is important for planning larger studies, as they highlight practical considerations for recruitment, retention, and engagement of participants. Despite these barriers, participants were willing to discuss sensitive issues in depth, demonstrating the feasibility of scaling up this research.

Also, the findings align with the National Multi-Sectoral Strategic Framework on HIV and AIDS (2021/22–2025/26), which emphasizes integrating mental health into HIV care, reducing stigma, and promoting awareness among PLWHA [30,31]. Hence, this study provides evidence to inform the implementation of these strategic plans and strengthen interventions addressing both HIV and mental health needs in Tanzania. By addressing these factors, an environment can be created where PLWHA with significant generalized anxiety symptoms feel empowered and supported to seek care, ultimately improving health outcomes.

### Recommendations

In line with the WHO Mental Health Gap Action Program (mhGAP), care for mental health among PLWHA experiencing significant generalized anxiety symptoms requires an approach that addresses both facilitators and barriers to treatment-seeking behaviors, such as creation of a psychosocial intervention that captures the improvement of awareness campaigns to reduce stigma and promote social support. Also, integration of mental health services into primary healthcare settings is essential to ensure early identification of significant generalized anxiety symptoms. These strategies support the WHO’s vision of closing the mental health treatment gap and ensuring that all individuals, including PLWHA, receive equitable, accessible, and effective mental health care.

### Study limitations

Recall bias is a limitation since it requires participants to remember information of anxiety symptoms from the past two weeks. This was mitigated by providing adequate time for response from the participants. The study was subject to difficulties in the retention of participants. This was mitigated by constant reminders to participants to attend mental health services by using phone calls, and those who were not able to attend were interviewed via phone calls to assess the barriers. We did not perform a comparative analysis between those retained and those lost to follow-up, which limits our ability to assess potential differences or biases introduced by attrition. Due to the nature of our study, there was an absence of inferential statistical analyses; as a result, the study cannot determine how demographic characteristics may influence treatment-seeking behaviors. Also, the study was conducted in only 2 of the 19 CTCs, lacked a control group, and had a small sample size, limiting the generalizability of the findings to the broader population of PLWHA. Another potential source of bias was social desirability, where participants might provide responses, they believe the researcher wants to hear. To minimize this in IDIs, the research team did not disclose their professional roles and emphasized that participants’ views were valuable, with no right or wrong answers. Facilitators used probing questions to encourage detailed responses. Additionally, multi-month dispensing of ARVs might have affected participants’ availability, potentially influencing the findings.

### Strength of the study

The study has addressed the mental health gap in the provision of services among the chronic diseases, such as HIV/AIDS, which can help to create an intervention potential for this vulnerable group. Also, the use of qualitative methods has captured personal experience that quantitative data might miss.

## Conclusion

While our findings provide preliminary insights into facilitators and barriers to mental health treatment-seeking among PLWHA with significant anxiety symptoms, they should be interpreted with caution due to the small-scale, exploratory nature of this pilot study. Future research should focus on larger, more representative studies to validate these findings and examine causal relationships. Additionally, studies could explore the effectiveness of integrating mental health services into CTCs and the impact of interventions targeting stigma, financial barriers, service accessibility, and awareness. Such research would provide stronger evidence to inform mental health policy and optimize service delivery for PLWHA in resource-limited settings.

## Supporting information

S1 Checklist Strobe checklist. (DOCX)

S1 File CRERC. (PDF)

S2 File Data. (CSV)
