# Supplementary material for: An exploratory qualitative pilot study assessing treatment-seeking behavior for generalized anxiety symptoms among people living with HIV/AIDS in Tanzania
Source: PLOS Ment Health. 2025 Oct 6;2(10):e0000348. doi: 10.1371/journal.pmen.0000348 (PMC12798625; doi:10.1371/journal.pmen.0000348)
Supplement: S1 File — (PDF) [file pmen.0000348.s002.pdf]

KILIMANJARO CHRISTIAN MEDICAL UNIVERSITY COLLEGE

(A Constituent College of Tumaini University Makumira)

P. O. Box 2240, MOSHI, Tanzania.

RESEARCH ETHICAL CLEARANCE CERTIFICATE

No. 2712

Research Proposal No. 1512

Study Title: A pilot study of assessing treatment-seeking behaviors for significant generalized anxiety symptoms among people living with HIV/AIDS in Kilimanjaro region: A prospective cohort study.

Study Area: CTCs – KCMC and Mawenzi Regional Referral Hospital

PI's Name: Frank Kiwango

Coinvestigators: Carl Mhina, Editruda Gamassa, Neema Allen and Florian Ghaimo

Institution (s): Kilimanjaro Christian Medical University College

The Proposal was approved by CRERC on: 3rd September, 2024

Duration of Study: One year

From: 3rd September, 2024 to 2nd September, 2025

PROF. MRAMBA NYINDO
Chair – CRERC

PROF. EPHATA KAAYA
Provost - KCMU College
